# Midventricular Takotsubo Cardiomyopathy Following COVID-19 Infection: Diagnostic Role of Cardiac Magnetic Resonance Tissue Mapping

**DOI:** 10.1016/j.cjco.2024.11.016

**Published:** 2024-11-26

**Authors:** Yoshito Kadoya, Bethlehem Mengesha, Luc Michel Beauchesne, Aun Yeong Chong, Marino Labinaz, D. Ian Paterson

**Affiliations:** Division of Cardiology, University of Ottawa Heart Institute, University of Ottawa, Ottawa, Ontario, Canada

**Takotsubo cardiomyopathy is typically characterized by reversible apical left ventricular (LV) akinesis, while atypical phenotypes involve the mid or basal segments. We describe the case of a 49-year-old woman with** **viral gastroenteritis and decompensated heart failure. Following normal coronary angiogram,** **an** **echocardiogram showed severe LV hypokinesis with circumferential dyskinesis in the midsegments consistent with atypical Takotsubo cardiomyopathy. Subsequent cardiac magnetic resonance (CMR) imaging revealed markedly improved LV function with diffuse myocardial edema in** **the** **mid and apical segments on tissue characterization. Our case highlights that CMR tissue mapping more accurately detects the full extent of myocardial injury in Takotsubo cardiomyopathy.**

A 49-year-old woman presented with recurrent vomiting, diarrhea, and low-grade fever of 2-days' duration and was diagnosed with a viral gastroenteritis. She was given intravenous fluids for dehydration; however, she subsequently developed significant dyspnea and hypoxia requiring noninvasive positive airway pressure ventilation. Laboratory tests showed an elevated troponin-T of 403 ng/L (normal < 14 ng/L) and nasal swab testing for SARS-CoV-2 was positive. A chest X-ray film showed pulmonary edema. Electrocardiogram revealed sinus tachycardia with diffuse T-wave flattening. Echocardiography revealed moderate to severe LV dysfunction with an ejection fraction (EF) of 31%. There was global LV hypokinesis with circumferential dyskinesis in the mid LV segments **(**Videos 1 and 2, view video online**)**. She subsequently underwent urgent coronary angiography, which revealed no epicardial coronary artery stenosis. She was diagnosed with congestive heart failure secondary to possible COVID-19-related myocardial injury. Two days later, CMR imaging demonstrated a markedly improved LV function (EF 52%) with mild hypokinesis of the midventricular segments **(**Videos 3 and 4 , view video online**)**. Tissue characterization imaging revealed possible nonischemic septal scar but no myocardial infarction on late gadolinium enhancement imaging **(**Fig. 1 and Supplemental Fig. S1**)**. T1 and T2 mapping showed increased values in the mid and apical LV segments consistent with myocardial edema (Fig. 2). Given rapid recovery of LV function and the circumferential midventricular wall motion abnormality, the patient was diagnosed with midventricular Takotsubo cardiomyopathy. A follow-up echocardiogram at 1 month revealed fully recovered regional and global LV function **(**Videos 5 and 6 , view video online**)**.

**Figure 1:**
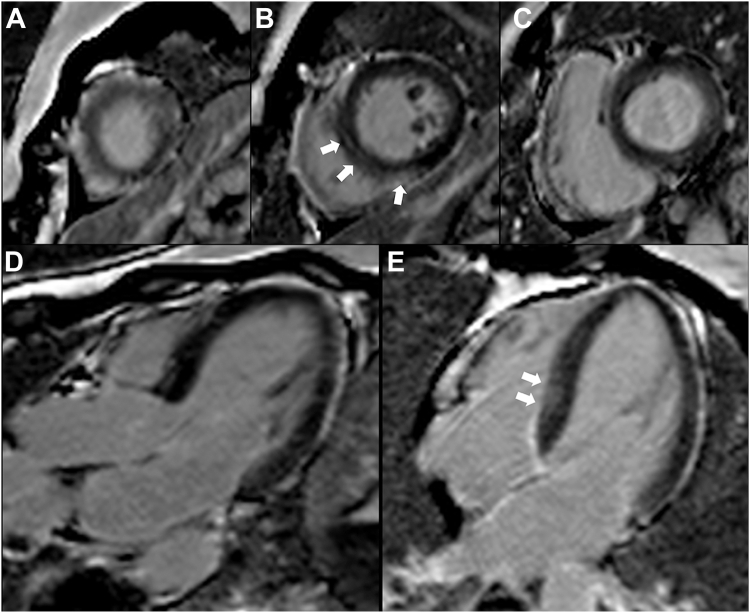
Late gadolinium enhancement sequence on cardiac magnetic resonance imaging showing possible focal mid wall (nonischemic) scar in the septum. (**A-C**) Short-axis view. (**A**) apical, (**B**) mid, and (**C**) basal segments. (**D**) Three-chamber view. (**E**) Four-chamber view.

**Figure 2:**
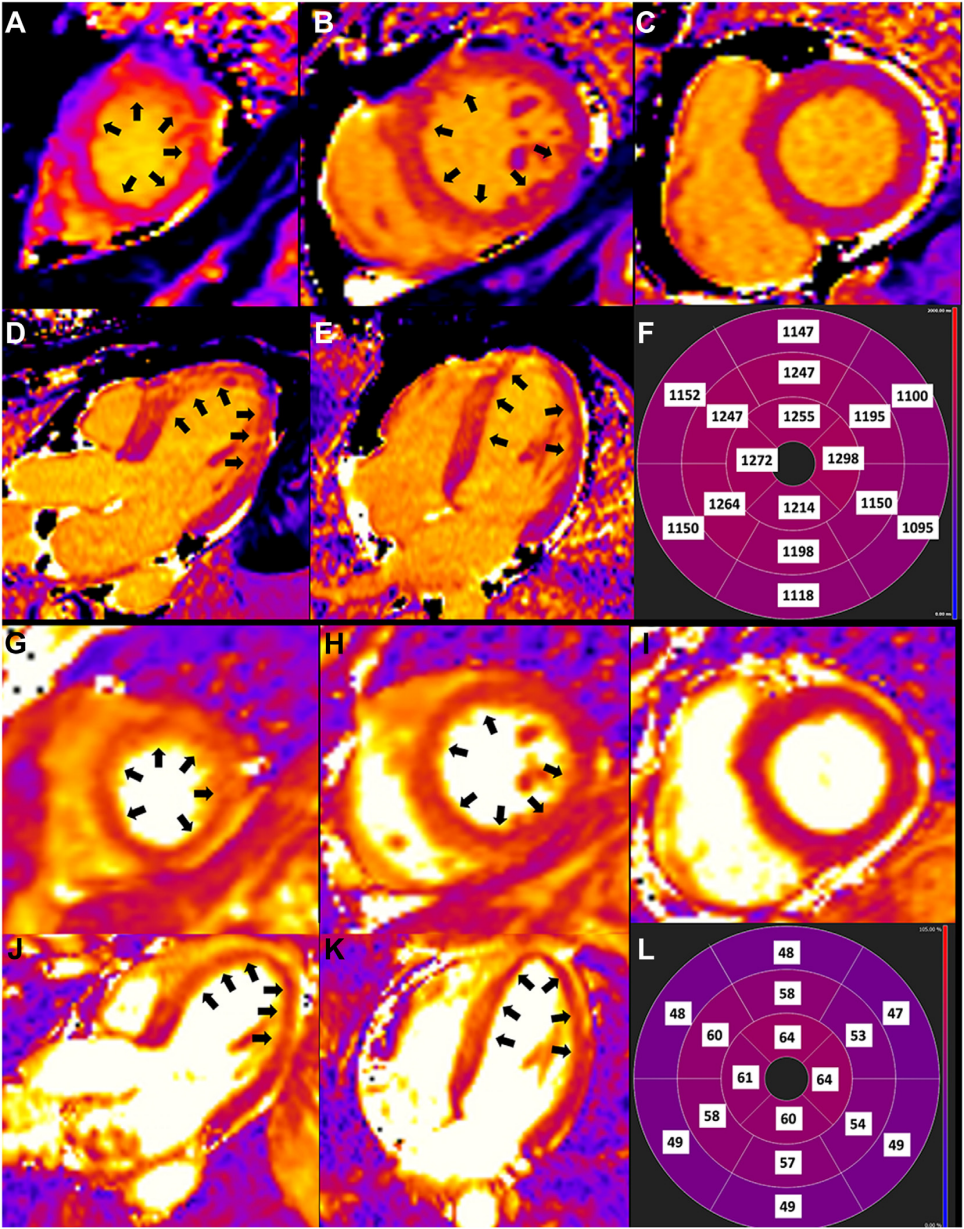
Myocardial T1 and T2 mapping on cardiac magnetic resonance imaging showing diffuse myocardial edema. (**A-F**) T1 mapping with native T1 value of 1150 to 1298 ms in the basal to apical ventricular segments (**black arrows**). Normal myocardial T1 < 1040 ms. (**A-C**)_Short-axis view. (**A**) Apical, (**B**) mid, and (**C**) basal segments. (**D**) Three-chamber view. (**E**) Four-chamber view. (**F**) Polar map with segmental native T1 values. (**G-L**) T2 mapping with T2 value of 57 to 64 ms in the affected mid to apical ventricular segments (**black arrows**). Normal myocardial T2 < 55 ms. (**G-I**) short-axis view. (**A**) Apical, (**B**) mid, and (**C**) basal segments). (**J**) Three-chamber view. (**K**) Four-chamber view. (**L**) Polar map with segmental T2 values.

## Discussion

COVID-19-related cardiac injury is highly prevalent in severe infection and is associated with a worse prognosis.^1^ Proposed mechanisms are poorly understood but can include viral cytopathic effects, secondary injury from systemic inflammation and hypoxemia, and vascular injury with thrombosis.^1^ Myocarditis related to COVID-19 infection or mRNA vaccines have been extensively reported; however, Takotsubo cardiomyopathy is less well recognized.

Takotsubo cardiomyopathy typically mimics an acute coronary syndrome and presents with chest pain syndrome following physical or emotional stress. Large regional wall motion abnormalities are commonly seen in the LV apex (apical ballooning); however, atypical phenotypes, including circumferential midventricular involvement is observed in 10% to 20% of patients. This less common phenotype has been associated with severe reductions in left ventricular ejection fraction (LVEF), cardiogenic shock, and concomitant heart failure.^2^ Despite atypical Takotsubo cardiomyopathy having more severe disease at presentation, long-term prognosis is reportedly comparable with the more common apical phenotype.^3^ The biological and clinical factors giving rise to atypical Takotsubo cardiomyopathy are not well understood, including the potential influence of COVID-19 infection.

In our case, the diagnosis was initially challenging because of the patient's unusual presenting symptoms and the atypical wall-motion abnormalities. However, CMR played a crucial role in differentiating Takotsubo cardiomyopathy from other COVID-19–related cardiac diseases, including myocarditis and myocardial infarction. Our case appeared most consistent with Takotsubo cardiomyopathy, although concurrent myocarditis cannot be excluded given the midseptal nonischemic enhancement. Furthermore, T1 and T2 tissue mapping identified the mid and apical LV involvement, more extensive than the wall-motion abnormalities. A CMR study of Takotsubo cardiomyopathy has shown increased myocardial T1 and T2 in areas of wall-motion abnormalities but did not evaluate segments with normal function.^4^ In our case, tissue-mapping abnormalities were evident in segments with recovered function and aligned with a more typical presentation of Takotsubo cardiomyopathy. Although Takotsubo cardiomyopathy phenotype has traditionally been based on the distribution of LV wall motion abnormalities, these findings are time sensitive. Our case highlights that tissue mapping more accurately detects the full extent of myocardial injury despite the delay in CMR. Current diagnostic criteria for Takotsubo cardiomyopathy includes a role for CMR to exclude myocarditis.^2^ However, this case illustrates the potential utility of myocardial tissue mapping to assist with Takotsubo cardiomyopathy subtyping in future updates.

**Novel Teaching Points**

• Multimodality imaging with echocardiography and CMR is useful to ascertain the etiology of myocardial injury with non-obstructive coronary artery disease.
• Compared to wall motion assessment, CMR derived tissue characterization imaging more accurately identifies the full extent of left ventricular involvement in patients with Takotsubo cardiomyopathy.

## Supplementary Material

Supplemental Figure 1

Video 1 Transthoracic echocardiogram apical 3-chamber view at presentation.

Video 2 Transthoracic echocardiogram apical 4-chamber view at presentation.

Video 3 Cardiac magnetic resonance imaging 3-chamber cine (balanced steady-state free precession cine sequence) at presentation.

Video 4 Cardiac magnetic resonance imaging 4-chamber cine (balanced steady-state free precession cine sequence) at presentation.

Video 5 Transthoracic echocardiogram apical 3-chamber view at 1 month.

Video 6 Transthoracic echocardiogram apical 4-chamber view at 1 month.
