# Supplementary material for: Midventricular Takotsubo Cardiomyopathy Following COVID-19 Infection: Diagnostic Role of Cardiac Magnetic Resonance Tissue Mapping
Source: CJC Open. 2024 Nov 26;7(2):141–4. doi: 10.1016/j.cjco.2024.11.016 (PMC11886365; doi:10.1016/j.cjco.2024.11.016)
Supplement: Supplemental Figure 1 [file mmc1.pdf]

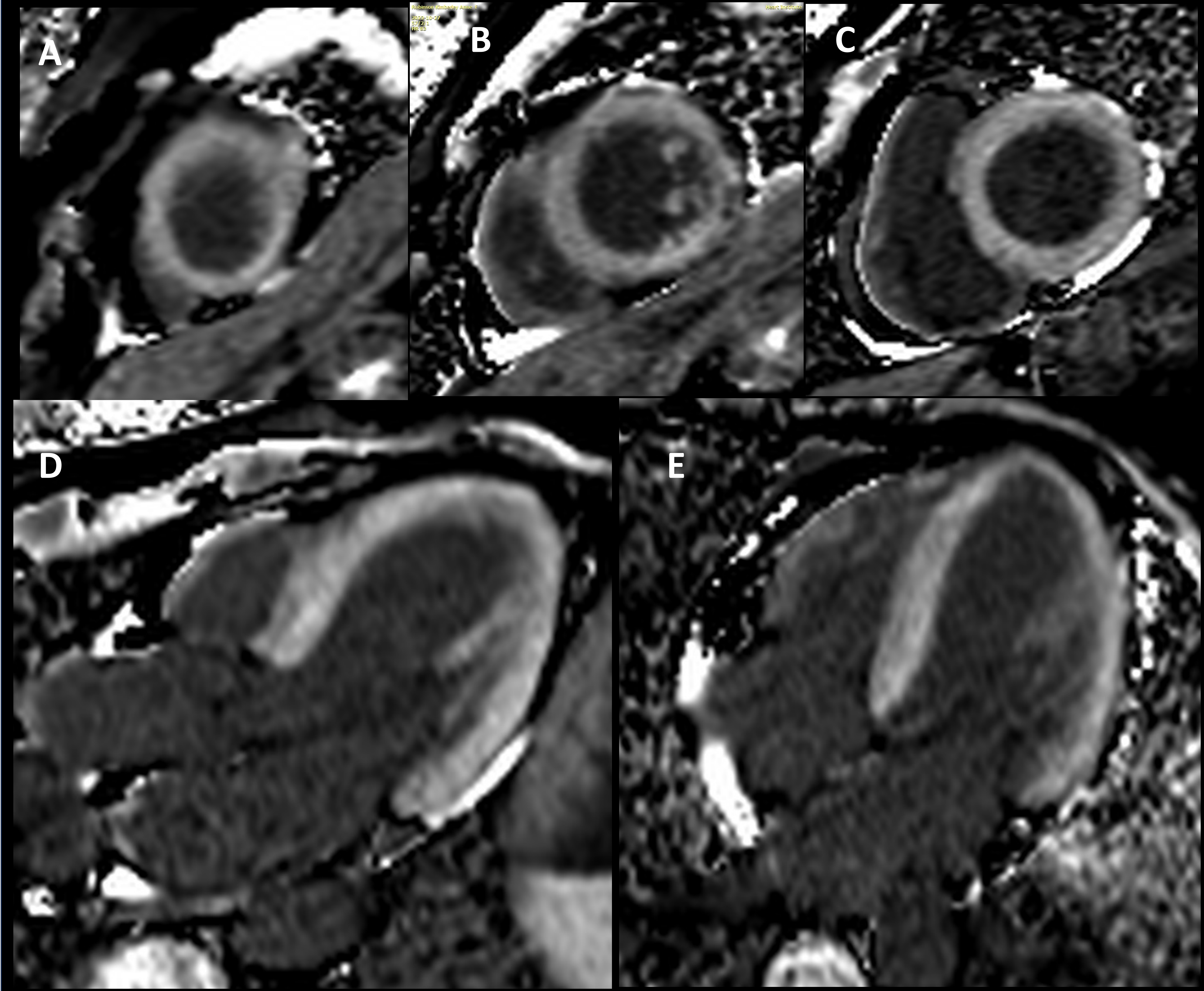

Supplemental Figure S1. Post-contrast T1 mapping images
A-C: short-axis view (A: apical, B: mid, and C: basal segments)
D: three-chamber view
E: four-chamber view
